# Variations by sex and age in the association between alcohol use and depressed mood among Thai adolescents

**DOI:** 10.1371/journal.pone.0225609

**Published:** 2019-12-17

**Authors:** Wit Wichaidit, Nannapat Pruphetkaew, Sawitri Assanangkornchai

**Affiliations:** 1 Epidemiology Unit, Faculty of Medicine, Prince of Songkla University, Hat Yai, Songkhla, Thailand; 2 Centre for Alcohol Studies, Hat Yai, Songkhla, Thailand; KHANA, CAMBODIA

## Abstract

**Background:**

Alcohol is associated with depression among adolescents, but variations in the association by age and sex are relatively unexplored. This study aims to assess variations in the association between alcohol consumption and depressed mood among adolescents by age and sex.

**Methods:**

We analyzed data from a school-based survey of 38,186 students in junior high school (Year 7 and 9) and senior high school (Year 11). The mean age of the participants was 15.2 (SD = 1.9) years. We used multivariate logistic regression to measure the association between self-reported alcohol drinking (past-year, past-month, and binge-drinking) and history of depressed mood for two weeks or more during the past year. We stratified the analyses by school level (as proxy for age group) and sex of the respondent.

**Results:**

Approximately 1% of students in surveyed schools refused to answer the questionnaires and fewer than 5% of all questionnaires were invalid. Prevalence of depressed mood was 13.2%. Prevalence of past-year alcohol drinking was 41.0% among those with depressed mood vs. 24.6% among those with no depressed mood (Adj OR = 1.78, 95% CI = 1.60, 1.98). The association was strongest among girls in junior high school (Adj OR = 2.38, 95% CI = 2.03, 2.79) and weakest among boys in senior high school (Adj OR = 1.19, 95% CI = 0.99, 1.42).

**Conclusion:**

Associations between alcohol drinking and depressed mood were particularly strong among junior high school girls. Youth mental health and alcohol programs should consider prioritizing this sub-group accordingly.

## Introduction

Depressive disorder is a medical illness that causes feelings of sadness and/or loss of interest in activities with symptoms lasting at least two weeks[1]. Depressive disorder is the most common cause of years lost to disability (YLD) among population aged 10–14 years old[2]. Depressive disorder is strongly associated with suicide[3], which is the third most common cause of death among adolescents[2]. The prevalence of depressive disorder seems to be higher among women[4] and in low and middle-income countries[3].

Alcohol is the most commonly used substance among adolescents in the Western world: among students aged 15–16, the prevalence of having five or more drinks (binge drinking) within last 30 days varied from 11% in the United States to 56% in Denmark[5]. In Thailand, the prevalence of binge-drinking among adolescents within the past 30 days is low (4%), but the prevalence of past-year drinking (26%) is between that of the United States and Denmark[6].

Thailand is a middle-income country in South East Asia. Common reasons mentioned for drinking among Thai drinkers are to socialize with one's peers, as well as a way to cope with problems faced by daily life [7]. Depression has been under-recognized in Thai society but is now frequently mentioned in the Thai media [8–10]. The proportion of Thais who received screening for mental health issues and mental health education increased from 4.2% in 2009 to 20.6% in 2016, and the proportion of those with depressive disorder who accessed mental health services increased from 5.1% to 48.5% during the same period[11]. A cross-sectional study of patients receiving outpatient treatment for depression at tertiary hospitals in Thailand showed that nearly 75% were women, the median age was 45 years, and 80% attended the service under an insurance scheme[12]. Among adolescents, a study showed that 13% of secondary school students in Thailand reported lifetime history of depression, while 7% reported history of suicidal ideation and 5% reported suicide attempt[6]. Although the current legal drinking age is 20 years [13], a national survey showed that approximately 13% of Thai adolescents age 15–19 years drank alcohol within the past 12 months[14].

Multiple studies have found that adolescent binge-drinking has negative long-term effects, including engaging in other risky behaviors (substance use, high-risk sexual contacts) and depression, which can present as early as during late adolescence and early adulthood[15–18]. Adolescents with depression have concurrent comorbidity with disruptive and emotional disorders, including increased risk for alcohol problems[4].

A systematic review of the literatures showed that alcohol use was associated with depression in adult population across multiple settings[19]. The association between alcohol use and depression may be particularly strong among younger adolescents[20], yet studies have not systematically assessed this variation. The association may also vary by gender: some studies found that the association between alcohol use and depression is higher among boys[16], although possibly confounded by higher prevalence of substance use comorbidity[20]. Other independent predictors of depression that could confound the association between alcohol and depression include family history of depression[3,4], exposure to psychosocial adversity and stressful life events[3,4,21], nature of living arrangements[21], parental behaviors[22], and psychiatric co-morbidities[21]. Furthermore, in general, adolescent girls are at higher risk of depression than adolescent boys[4]. Thus the role of gender in the association between alcohol consumption and depression also needs to be assessed. A study among adolescents seeking mental health services at community hospitals in northern Thailand also found association between depression, drinking behaviors, and risk of suicide, but did not assess effect modification by age and sex[23]. Description of variations in the association between alcohol use and depression between sex and age groups can identify the group where the association is strongest, and help to inform stakeholders with regard to allocation of resources and services accordingly.

Diagnosis of depressive disorder is a thorough process involving clinical interviews and possible physical examination[1]. However, tools such as the Patient Health Questionnaire-2 may contain as few as two questions (one on depressed mood, the other on anhedonia) and can be self-administered for rapid population-level screening for depressive disorder[24,25]. These screening tools are potentially useful for epidemiological studies. The objectives of this study are: 1) to assess the extent to which alcohol consumption is associated with depressed mood among Thai adolescents, and; 2) to assess the extent to which sex and age group modify the association between alcohol consumption and depressed mood.

## Materials and methods

### Study design and setting

The National School Survey on Alcohol Consumption, Substance Use and Other Health-Risk Behaviors was a cross-sectional survey conducted to provide information about magnitude and trend of alcohol consumption, tobacco use, illicit drug use and risky behaviors among Thai adolescents in Thailand’s formal educational system. The 2016 survey was conducted, as the third survey of its type, following the first two surveys in 2007 and 2009[6].

The survey included students in Year 7 (age 12–13 years), Year 9 (age 14–15 years), and Year 11 (age 16–17 years). Students in Year 7 and Year 9 (Matthayom 1 and Matthayom 3) are in a common general education junior high school system. Students in Year 11 attend either general education high school (Matthayom 5) or vocational school (Vocational Certificate, Year 2). The survey was not allocated to students in Year 8, Year 10, and Year 12. The survey included public and private schools in urban and rural areas (i.e., inside and outside of municipality areas as designated by the Thai Ministry of the Interior, respectively) in 40 out of 77 provinces of Thailand.

### Sampling method

The survey researchers used multi-stage cluster random sampling to recruit the participants. First, Thailand's 77 provinces were divided into 12 education regions. Second, half of the provinces in each education region were randomly selected, yielding 40 provinces. Third, in each province, 5 schools were selected: 1 urban-area general-education public school, 1 rural-area general-education public school, 1 private school (urban-area only; rural-area private schools were very uncommon), 1 commercial vocational school, and 1 technical vocational school. In Bangkok, 4 schools were selected (Bangkok had no rural public school). Fourth, classrooms were randomly selected. At schools with 5 or more classrooms per grade level, 3 classrooms were selected per grade level. At schools with fewer than 5 classrooms per grade level, all classrooms were selected. Fifth, we collected data from all students in the sampled classrooms.

### Data collection

Project coordinator in each province made a visit to the sampled school and presented a letter explaining the details of the study to the school administrators, and made an appointment for the date of data collection. The schools had the right to refuse participation. Before collecting data from each classroom, project coordinators briefed the students regarding the objectives and the methodology of the study, and that their participation was voluntary. Project coordinators also explained to the students about privacy and confidentiality, and requested the students to answer the questions truthfully. Project coordinators informed the students that the self-administered questionnaire would take approximately 20 min to complete. Students who agreed to participate would give verbal consent. The students then administered and completed the questionnaire, put the completed questionnaire in an individual envelope, and placed the envelope in a pile in front of the classroom. The coordinators then bound the envelopes from the same classroom together. Afterward, research staff collected the questionnaires and examined their completion and validity. Questionnaires where 30% or more of the questions were not answered, or the pattern of the answers indicated potential lack of intention to respond in a truthful manner, were excluded from the study as were those who did not answer the questions relevant for this study. Study staff then entered data from the remaining paper questionnaires into an electronic database and verified the validity of the electronic data. A waiver of the need for a document of consent for minors was approved from the institutional review board. As anonymity was guaranteed and research procedures entailed no more than minimal risk to subjects, providing consent by action *in lieu of* written informed consent did not affect the rights and welfare of the subjects.

In this survey, the participation rate was 99%: one percent (1%) of students refused within the schools that agreed to participate. Fewer than 5% of all questionnaires were incomplete or potentially invalid and excluded from the study.

### Measures

The questionnaire contained 6 sections: A) Student demographics and background characteristics; B) Household, school and community environments; C) Disciplinary actions from family and school; D) Smoking, alcohol consumption, illicit drug use, gambling, and sexual behaviors; E) Safety and violence; F) Gaming and social media use. Questions in this study, including those that measured adolescent drinking and depressed mood, were modified from the questionnaire used in the Surveillance of Drinking Behaviors and Other Health Risk Behaviors among High School Students in Thailand 2008[26] and a review of the related literature[6].

In Section A, student demographics and background characteristics included religion, geographic region, living situation, grade point average, and school type. In Section B, household, school and community environments included self-reported information on whether their parents or stepparents had "problems" with alcohol consumption, smoking, drug use, gambling, gaming addiction, and violence. In Section E, safety and violence issues included self-reported experience of being threatened, engaging in mild and severe physical violence, and experiences of intimate partner violence and sexual violence. We considered these experiences as exposure to psychosocial adversity and stressful life events and refer to them as "adverse experiences".

### Adolescent drinking

Experience of drinking alcohol was self-reported in a specific section pertaining to consumption of "alcoholic beverage" (defined as "drinks containing ethanol that causes intoxication upon consumption including whiskey, liquor, wine, beer, fruit punch, rice wine, and fermented sugar, etc."). The beginning of the alcohol section of the questionnaire included pictures of standard unit of alcoholic drinks. The questionnaire included whether participants had consumed alcohol in their lifetime, within the past 12 months, and within the past 30 days. Those who had consumed alcohol within the past 30 days were asked about the number of drinking sessions within the past month, units of alcohol consumed per drinking session, and number of sessions with drunkenness. Those who had consumed alcohol within the past month and had 5 or more standard drinks per “typical” drinking session (among girls) or 6 or more standard drinks per “typical” drinking session (among boys) were considered to have engaged in binge-drinking[27,28].

For consistency between measures of alcohol consumption, we decided to categorize the respondents as: 1) those who drank within the past 12 months; 2) those who drank within the past 30 days, and; 3) those who binge-drank within the past 30 days. Each categorization was treated as a separate outcome. We excluded missing data regarding drinking behaviors from the analyses.

### Depressed mood

The depressed mood question was modified from the depressed mood item of the Patient Health Questionnaire-2, which is used for detection of major depression in adults[24] and adolescents[25]. We asked participants about depression as one of a number of health risk behaviors in the Survey, thus we decided to reduce the measurement to only one question in the Safety and Violence section: “*In the past 12 months*, *have you ever felt depressed*, *despair*, *or down almost every day for a period of two weeks or more*, *to the point where you could not do your routine daily function*?”. Possible answers were “*1) No; 2) Yes*”. We considered students who answered “Yes” as those who had depressed mood, and “No” as those who did not have depressed mood. We excluded missing data (non-responses) from the analyses.

### Statistical methods

We first described the prevalence of demographic factors, drinking, and depressed mood among study participants, disaggregated by sex and year of study. We used weighting compensation techniques[29] to account for the complex survey design and estimated the national-level prevalence (with standard error values) for each of the measures.

Potential confounders that we assessed in the analyses included: geographic region, religion, school type, living situation, grade point average (GPA, on a 4.0 scale with 4 as the highest grade and 0 as the lowest), smoking, past-year adverse experiences, and having parent(s) with addiction or violence-related problems. We cross-tabulated potential confounder variables against history of depressed mood within the past year and assessed the statistical significance of the association using chi-square test of independence. We retained potential confounders that were associated with depressed mood at p-value < 0.15 for multivariate logistic regression, and calculated adjusted odds ratios with 95% confidence intervals.

We assessed effect modification by age (using school year as the proxy measure) and sex by including interaction terms in multivariate logistic regression models. We then stratified our analyses by levels of the potential effect modifiers whose interaction terms were statistically significant.

We divided students into 2 age groups: junior high school (Year 7 and 9) and senior high school / vocational college (Year 11 / Vocational Certificate Year 2). We grouped Years 7 and 9 together because the common age range of students in these levels were 11–14 years, which corresponds to early adolescence. Students in Year 11 / VC2 were generally 16–17 years of age, i.e., in middle adolescence. There were two questions to measure sex: one question asked about the sex at birth of the student (i.e., birth gender), and one question asked about the sex that the student identified (i.e., self-identified gender). Answers to the birth gender question had higher completeness (0.9% missing data) compared to the answers to the self-identified gender question (17.1% missing data), and we decided to use only the answers to the birth gender question as a measure of sex of the student.

All data analyses were conducted using the R with Epicalc[30] package. We also accounted for the complex survey design in our analyses with the Survey package for R[29].

### Ethical considerations

The 2016 national school survey was approved by Khon Kaen University Ethical Review Board for Research in Human Subjects (EC: 59-396-18-1, Project Number HE581430). Ethical clearance for secondary data analysis was obtained from the Ethics Committee for Research in Human Subjects of the Faculty of Medicine, Prince of Songkla University.

## Results

### Characteristics of study participants

The 2016 survey data included 195 schools and 38,186 participants (age mean ± SD = 15.2 ± 1.9 years), including 21,804 students in Matthayom 1 and 3 (M1 and M3; Year 7 and 9 in a 12-year educational system, i.e., junior high school; age mean ± SD = 12.8 ± 0.6 years and 14.8 ± 0.6 years, respectively), and 16,382 students in Mathayom 5 or Vocational Certificate 2 (M5 or VC2; Year 11 in a 12-year educational system, i.e., senior high school; age mean ± SD = 16.8 ± 0.6 years and 17.2 ± 1.1 years, respectively). Among the participants, 55.9% were female, 90.2% were Buddhists, 84.5% were living in the family's own home or apartment, and 99.1% were full-time students. With regard to smoking, 23.8% of male students and 6.6% of female students reported having smoked more than 5 packs of cigarettes during their lifetime.

### Prevalence of alcohol consumption and depressed mood

Approximately 26.6% of respondents had consumed alcohol within past 12 months, and 7.0% had done binge-drinking in past 30 days (Table 1). The prevalence of drinking in past 12 months, drinking in past 30 days, and binge-drinking in past 30 days were higher among boys than girls, and higher among older students (Year 11) compared to younger students (Years 7 and 9). Demographic characteristics were all similar between boys and girls, and between older students and younger students. Approximately 13.2% of study participants reported having depressed mood, and 9.0% reported history of suicidal ideation. Prevalence of depressed mood and suicidal ideation were slightly higher among girls than boys, and among older students compared to younger students.

**Table 1:** Prevalence of demographic factors, drinking, smoking, suicidal behaviors, and depressed mood among study participants.

	By sex			By year of study		
Characteristic	Boys (n = 17,381)	Girls (n = 20,805)	P-value^a^	Years 7 and 9 (n = 22,003)	Year 11 (n = 16,532)	P-value^a^
	Percent (SE)^b^	Percent (SE) ^b^		Percent (SE) ^b^	Percent (SE) ^b^	
Depressed mood (% yes)	12.3 (0.6)	13.9 (0.7)	0.006	11.6 (0.6)	15.2 (0.7)	<0.001
Suicidal ideation (% yes)	8.4 (0.6)	9.5 (0.6)	0.058	8.9 (0.6)	9.4 (0.7)	0.679
Drinking in past 12 months (% yes)	28.7 (1.0)	24.9 (1.4)	<0.001	19.2 (0.8)	35.0 (1.5)	<0.001
Drinking in past 30 days (% yes)	20.2 (0.9)	16.9 (1.0)	<0.001	13.2 (0.7)	24.2 (1.2)	<0.001
Binge-drinking in past 30 days (% yes)	7.7 (0.5)	6.4 (0.6)	0.035	4.6 (0.3)	9.8 (0.7)	<0.001
Smoking in past 12 months (% yes)	15.2 (1.2)	3.5 (0.7)	<0.001	8.7 (0.6)	14.4 (1.1)	<0.001
**Religion**						
Buddhism	89.9 (2.9)	90.4 (2.7)	0.748	89.8 (3.0)	90.3 (2.4)	0.161
Islam	7.6 (3.0)	7.0 (2.8)		7.8 (3.1)	6.7 (2.6)	
Christianity	2.0 (0.4)	2.3 (0.5)		2.0 (0.4)	2.4 (0.4)	
Others	0.5 (0.1)	0.3 (0.1)		0.4 (0.1)	0.6 (0.1)	
**Region**						
Special-Bangkok	8.2 (7.8)	11.1 (10.2)	0.636	8.5 (8.0)	11.4 (10.4)	0.088
Bangkok Metro Areas	5.2 (3.7)	4.2 (3.1)		4.7 (3.3)	4.7 (3.4)	
Central	22.0 (7.1)	21.6 (7.4)		21.5 (7.1)	21.8 (7.4)	
South	15.2 (6.6)	13.9 (5.9)		15.3 (6.4)	13.5 (5.8)	
North	18.8 (6.6)	19.4 (7.1)		18.9 (6.7)	19.6 (7.0)	
Northeast	30.6 (8.7)	29.8 (8.9)		31.1 (8.8)	29.1 (8.7)	
**Living Situation**						
Family home/flat	84.3 (2.0)	84.8 (1.6)	0.241	85.2 (2.1)	83.5 (1.4)	<0.001
School dorm	4.4 (2.1)	3.4 (1.2)		4.1 (1.9)	3.5 (1.2)	
Outside dorm	3.1 (0.5)	2.7 (0.4)		1.4 (0.3)	4.6 (0.7)	
Rented home	6.7 (1.1)	7.5 (1.4)		7.5 (1.4)	6.7 (1.1)	
Others (relatives, temple)	1.6 (0.2)	1.7 (0.3)		1.8 (0.2)	1.7 (0.3)	
**Grade point average (GPA)**						
GPA = 0.1–1.0	0.7 (0.1)	0.1 (0.0)	<0.001	0.4 (0.1)	0.3 (0.1)	<0.001
GPA = 1.1–2.0	11.5 (0.9)	3.8 (0.4)		8.1 (0.8)	6.1 (0.6)	
GPA = 2.1–3.0	44.7 (1.1)	34.0 (1.4)		35.6 (1.3)	42.4 (1.3)	
GPA = 3.1–4.0	33.8 (1.8)	55.0 (1.9)		47.6 (2.1)	43.4 (1.8)	
Unknown	9.3 (0.8)	7.1 (0.8)		8.3 (0.8)	7.8 (0.7)	
**School type**						
Government	67.4 (2.7)	66.8 (3.2)	0.873	67.3 (2.6)	66.6 (4.5)	0.873
Private	31.4 (2.8)	31.9 (3.3)		31.3 (2.7)	32.2 (4.6)	
Unknown	1.2 (0.3)	1.3 (0.5)		1.4 (0.4)	1.2 (0.4)	
**Past-year adverse experiences**						
Being threatened (% yes)	7.7 (0.5)	2.0 (0.1)	<0.001	3.5 (0.2)	5.7 (0.5)	0.004
Severe physical violence (% yes)	7.6 (0.4)	2.7 (0.2)	<0.001	4.0 (0.2)	5.8 (0.4)	0.010
Intimate partner violence (% yes)	7.0 (0.3)	3.1 (0.3)	<0.001	3.7 (0.2)	6.0 (0.4)	0.001
Sexual violence (% yes)	3.0 (0.3)	1.3 (0.1)	<0.001	1.3 (0.1)	3.0 (0.3)	0.001
Had any adverse experience in past year (% yes)	14.8 (0.9)	5.8 (0.3)	<0.001	7.6 (0.3)	12.2 (0.1)	0.002
**Parents’ addiction or violence problem(s)**						
Had a father with one or more problem(s) (% yes)	3.6 (0.3)	3.5 (0.3)	0.714	3.5 (0.3)	3.5 (0.3)	0.964
Had a mother with one or more problem(s) (% yes)	2.4 (0.3)	2.2 (0.2)	0.404	2.2 (0.2)	2.4 (0.3)	0.313
Had a stepmother with one or more problem(s) (% yes)	0.3 (0.0)	0.4 (0.1)	0.894	0.4 (0.0)	0.3 (0.1)	0.796
Had a stepfather with one or more problem(s) (% yes)	0.6 (0.1)	0.7 (0.1)	0.188	0.5 (0.1)	0.7 (0.1)	0.177
Had at least one parent with one or more problem(s) with addiction or violence (% yes)	5.7 (0.5)	5.7 (0.3)	0.803	5.7 (0.4)	5.8 (0.5)	0.931

^a^For Chi-square test of independence between groups in the two preceding columns

^b^Estimated prevalence (standard error)

### Association between alcohol consumption and depressed mood

Depressed mood was significantly associated with drinking in past 12 months (41.0% among students with depressed mood vs. 24.6% among students with no depressed mood), drinking in past 30 days (29.1% vs. 16.9%), and binge-drinking in past 30 days (11.3% vs. 6.3%). Other co-variables associated with depressed mood included smoking, being in the older class, being female, being from Bangkok, having had an adverse experience in the past year, and having at least one parent with problems with addiction or violence (Table 2).

**Table 2:** Distribution of participants with and without depressed mood by associated factors.

Characteristic	No Depressed Mood (n = 31,107) %(SE^b^)	Depressed Mood (n = 4,594) %(SE^b^)	P-value^a^
**Drinking in past 12 months**			
No	75.4 (0.9)	59.0 (1.6)	<0.001
Yes	24.6 (0.9)	41.0 (1.6)	
**Drinking in past 30 days**			
No	83.1 (0.8)	70.9 (1.4)	<0.001
Yes	16.9 (0.8)	29.1 (1.4)	
**Binge-drinking in past 30 days**			
No	93.7 (0.5)	88.7 (0.9)	<0.001
Yes	6.3 (0.5)	11.3 (0.9)	
**Smoking**			
No	89.9 (0.7)	82.6 (1.6)	<0.001
Yes	10.1 (0.7)	17.4 (1.6)	
**Year Level**			
Years 7 & 9	54.8 (0.9)	47.1 (1.3)	<0.001
Year 11 & Vocational 2	45.2 (0.9)	52.9 (1.3)	
**Sex**			
Female	56.4 (1.2)	59.8 (1.6)	0.006
Male	43.6 (1.2)	40.2 (1.6)	
**Religion**			
Buddhism	90.4 (2.8)	90.4 (2.5)	0.161
Islam	7.4 (2.9)	6.5 (2.7)	
Christianity	2.1 (0.4)	2.7 (0.6)	
Other	0.1 (0.0)	0.4 (0.1)	
**Region**			
Special-Bangkok	9.3 (8.7)	13.8 (12.2)	0.013
Bangkok Metro Areas	4.7 (3.3)	5.1 (3.6)	
Central	21.2 (7.1)	23.2 (8.1)	
South	14.3 (6.1)	13.2 (5.7)	
North	20.7 (7.2)	17.4 (6.7)	
Northeast	29.8 (8.6)	27.3 (8.7)	
**Living situation**			
Family home/flat	85.2 (1.7)	83.2 (2.0)	0.147
School dorm	3.8 (1.7)	3.7 (1.7)	
Outside dorm	2.9 (0.4)	3.3 (0.5)	
Rented home	6.9 (1.2)	8.4 (1.4)	
Others (relatives, temple)	1.2 (0.2)	1.4 (0.3)	
**Grade point average (GPA)**			
GPA = 0.1–1.0	0.3 (0.1)	0.7 (0.1)	0.061
GPA = 1.1–2.0	7.1 (0.5)	7.6 (0.9)	
GPA = 2.1–3.0	38.8 (1.2)	38.2 (1.3)	
GPA = 3.1–4.0	45.7 (1.8)	44.9 (2.0)	
Unknown	8.0 (0.8)	8.5 (1.2)	
**School type**			
Government	67.2 (2.9)	63.0 (3.4)	0.092
Private	31.6 (3.0)	35.7 (3.8)	
Unknown	1.1 (0.3)	1.2 (0.5)	
**Had any adverse experience in past year**			
No	92.1 (0.4)	78.4 (1.1)	<0.001
Yes	7.9 (0.4)	21.6 (1.1)	
**Had at least one parent with one or more problem(s) with addiction or violence**			
No	95.0 (0.3)	89.8 (0.8)	<0.001
Yes	5.0 (0.3)	10.2 (0.8)	

^a^For Chi-square test of independence

^b^Standard Error

After adjusting for region, religion, school type, living situation, GPA, smoking, past-year adverse experience and having at least one parent with addiction or violence problems, students who reported drinking in the past year (12 months) and in the past month (30 days) had significantly higher odds of depressed mood than students who did not report drinking (Table 3). Students who reported binge-drinking in past 30 days also had about 48% higher odds of depressed mood than those who did not. Tests of statistical interactions with sex and age (year level) were all statistically significant (p-value < 0.05).

**Table 3:** Association between alcohol consumption behaviors and depressed mood.

Drinking behavior	Depressed Mood OR (95% CI)^a^^,^^b^	
	Model 1^c^	Model 2^d^
**All study participants**		
Past-year drinking (Ref.: No drinking in past year)	**2.12 (1.98, 2.28)**	**1.78 (1.60, 1.98)**
Past 30 days drinking (Ref.: No drinking in past 30 days)	**2.03 (1.89, 2.17)**	**1.65 (1.51, 1.82)**
Binge-drinking in past 30 days (Ref.: No binge-drinking in past 30 days)	**1.90 (1.66, 2.17)**	**1.48 (1.28, 1.70)**
**Among girls in Years 7 & 9**		
Past-year drinking (Ref.: No drinking in past year)	**2.88 (2.51, 3.31)**	**2.38 (2.03, 2.79)**
Past 30 days drinking (Ref.: No drinking in past 30 days)	**2.87 (2.46, 3.36)**	**2.35 (2.05, 2.69)**
Binge-drinking in past 30 days (Ref.: No binge-drinking in past 30 days)	**2.57 (2.08, 3.17)**	**1.97 (1.55, 2.51)**
**Among boys in Years 7 & 9**		
Past-year drinking (Ref.: No drinking in past year)	**2.21 (1.79, 2.74)**	**1.74 (1.43, 2.11)**
Past 30 days drinking (Ref.: No drinking in past 30 days)	**2.13 (1.69, 2.69)**	**1.59 (1.29, 1.97)**
Binge-drinking in past 30 days (Ref.: No binge-drinking in past 30 days)	**2.19 (1.66, 2.88)**	**1.61 (1.17, 2.22)**
**Among girls in Year 11 & Vocational 2**		
Past-year drinking (Ref.: No drinking in past year)	**1.97 (1.69, 2.30)**	**1.65 (1.34, 2.03)**
Past 30 days drinking (Ref.: No drinking in past 30 days)	**1.90 (1.72, 2.11)**	**1.54 (1.33, 1.79)**
Binge-drinking in past 30 days (Ref.: No binge-drinking in past 30 days)	**1.85 (1.48, 2.31)**	**1.42 (1.15, 1.75)**
**Among boys in Year 11 & Vocational 2**		
Past-year drinking (Ref.: No drinking in past year)	**1.43 (1.17, 1.75)**	1.19 (0.99, 1.42)
Past 30 days drinking (Ref.: No drinking in past 30 days)	**1.35 (1.07, 1.69)**	1.10 (0.89, 1.37)
Binge-drinking in past 30 days (Ref.: No binge-drinking in past 30 days)	1.23 (0.94, 1.60)	1.04 (0.80, 1.34)

^a^Odds Ratio with 95% confidence interval

^b^P-values < 0.05 for all interaction terms between exposure (drinking behavior) and sex and school level

^c^Model 1: Crude (unadjusted) OR (95% CI)

^d^Model 2: OR (95% CI), adjusted for region, religion, school type, living situation, grade point average (GPA), smoking, past-year adverse experience and having at least one parent with addiction or violence problems

Associations between alcohol consumption and depressed mood varied across sex and age groups. Stratified analyses showed that after adjusting for potential confounders, the association between past year-drinking and depressed mood was strongest among girls in Years 7 and 9 (junior high school students) (Adjusted OR = 2.38, 95% CI = 2.03, 2.79) compared to boys in Years 7 and 9 (Adjusted OR = 1.74, 95% CI = 1.43, 2.11), girls in Year 11 (Adjusted OR = 1.65, 95% CI = 1.34, 2.03), and boys in Year 11 (Adjusted OR = 1.19, 95% CI = 0.99, 1.42) (Table 3). Similarly, the associations between past-month drinking / binge-drinking and depressed mood were strongest among female students in Years 7 and 9, and weakest among boys in Year 11.

## Discussion

Our analyses of data from a national cross-sectional survey on risky behaviors and mental health of school adolescents showed that alcohol consumption was associated with depressed mood among Thai adolescents, and that age and sex modified this association. The association between drinking and depressed mood was strong in all groups, but it was strongest among early-adolescence girls (those in Year 7 and Year 9).

Compared to results from a national survey among youths aged 15–24 years[14], the prevalence of past-year, past-month and binge-drinking among girls in this study were higher than the national average, while such prevalences among boys were lower than the national average. The prevalence of depressed mood among adolescents in our study was lower than the prevalence of depression in studies among school students in Northeast Thailand[31] but higher than the prevalence in a cross-sectional study among Year 11 students in Bangkok[32]. However, each of these studies used different tools to measure adolescent depression, which limited the comparability of the findings. The study among Year 11 students in Bangkok showed that pessimistic thinking toward self, dissatisfaction with grades, and parent-child relationship problems were most strongly associated with depression[32]. The association between depression and alcohol could have been mediated by these factors, or drinking may be used by Thai adolescents as a coping mechanism against the risk factors.

Interpretation of the study findings should take into account the measurement of alcohol consumption and how the Survey measured depressed mood. The depression screening question is based on one of the two items that constituted the Patient Health Questionnaire-2 (PHQ-2) screening tool[24]. However, the PHQ-2 measured ongoing depressed mood within past two weeks and asked the respondent to indicate the frequency of depressed mood. Our study instrument asked whether the respondent had experienced depressed mood almost daily for two weeks or more within the past year. Those who answered "*Yes*" to our depressed mood question would likely score ≥3 on the PHQ-2, which had a sensitivity of 74% and specificity of 75% for detecting major depression according to DSM-IV criteria[25]. Adolescents with depression could be among those classified in this study as “no depressed mood”, and vice versa. The use of both PHQ-2 items could further improve the positive predictive value of the assessment[25] and should be considered for future studies on depression among Thai adolescents[33]. Furthermore, measurement of binge-drinking was defined by number of drinks per one "typical" drinking session during the past month, and not the more common definition of whether a participant had consumed 5 or more standard drinks in one session at any point within the past month[34], affecting the comparability of our study findings with regard to the association between binge-drinking and possible depression. Lastly, there is potential for residual confounding in having at least one parent with problems with addiction or violence. In Thai language, the word "problem" in the context of smoking and drinking could be interpreted not as substance abuse (e.g., tobacco or alcohol use disorder), but simply as whether the parent was a current smoker or a current drinker (regardless of whether there was a use disorder). A survey in 2017 showed that the majority of the Thai population did not drink, and those who drank typically did so in moderate amount[14]. We found that inclusion of problems with tobacco and alcohol consumption grossly over-estimated the number of problem drinkers compared to the survey[14], and so we decided not to consider "problem" with smoking or alcohol consumption as problems with addiction or violence. As a consequence, residual confounding from having a parent with alcohol use disorder might have been present in the findings. Having a parent with problems related to addiction or violence was positively associated with depressed mood, thus residual confounding could have led to over-estimated measures of association between drinking behaviors and depressed mood.

The findings of this study suggested that age and sex can jointly modify the effect of alcohol on depressed mood. Effect modification by age and sex in this study is supported by the body of literature[15–18,35]. In a cross-sectional study in Norwegian adolescents, depressive symptoms were associated with earlier onset of alcohol use, more frequent consumption and intoxication, and these associations were stronger for girls than boys[36]. Difference by gender in the association between depression and alcohol use was also found in a longitudinal study among middle-school students in the United States[37]. Adolescents at different ages have different neurological responses to alcohol exposure, and adolescent girls are at higher risk of depression than adolescent boys[16]. Prevalence of depression positively correlates with age during adolescence[38], and a review of observational studies on the etiology of depression suggested a developmental stage and sex-specific protective mechanisms[39]. Furthermore, in describing effect modification by sex, we did not consider the students’ self-identified gender, and thus transgender students were not identified nor considered as a separate group. Fewer than 2% of students who answered both the birth gender and self-identified gender questions were transgender. However, as 17% of the survey respondents did not answer the self-identified gender question, the number of transgender students could have been under-estimated in this study. Although a recent survey among Thai transgender adolescents showed high-level of self-reported quality of life[40], transgender adolescents also suffer from multiple abuses at the hand of their peers and the larger society with little indication of systematic legal protection[41]. The existing classification of sex and gender also did not allow for identification as a gender non-binary person[42]. Future studies should consider more complete and systematic identification of gender status and report the findings for adolescents who are not transgender and gender non-binary.

There are a number of public health implications and suggestions for future studies based on our study’s findings. Associations between alcohol consumption and depressed mood were particularly strong among junior high school girls, which imply that youth mental health services that serve early adolescent girls with depression should monitor their clients with regard to alcohol use, and youth alcohol prevention and control programs that work with early adolescent girls should monitor the mental health of their target population. In this study, the function of alcohol consumption in depression, and the function of depression in drinking behavior, were not explored. Future studies should consider qualitative investigation to understand adolescent experiences of drinking and depression, particularly among early adolescent girls. Because of the cross-sectional study design, we could not assess the long-term effect of alcohol consumption. Studies have found that drinking frequency at age 13–15 years was associated with prevalent depression 4 years later[16], and that the prevalence of depression and alcohol-use disorder (AUD) co-morbidity increased substantially from adolescence to young adulthood[43,44]. Future studies should consider prospective cohort study design and, if possible, follow the participants into young adulthood. Lastly, vocational students in Thailand are generally more likely than general education students to engage in risky behaviors including street racing, fighting, drinking and drug use[45,46]. These risky behaviors may have stemmed from adverse childhood experiences, which increases the susceptibility to depression. Vocational college students who drink may be more susceptible to depression than general education students who drink. Future studies should include an assessment of whether the association between alcohol consumption and depressed mood is stronger among vocational students than general education students.

The study has several strengths: The survey was conducted in a systematic manner in large and nationally-representative samples. This study was the first to assess the association between three types of drinking behaviors and depressed mood, and variations in this association by sex and age among Thai adolescents. However, the study also has a number of limitations. Firstly, the cross-sectional design of this study did not allow for causal inferences, and reverse causality was possible as a previous study found association between depression and increased risk for alcohol problems[4] as well as bi-directional association between alcohol and depression[37]. A more cautious interpretation would be that adolescents with depressive symptoms can potentially develop alcohol use behavior, and adolescents who drink can potentially develop depressive symptoms. Compared to other groups, younger adolescent girls with depressive symptoms have higher odds of alcohol use behavior, and younger adolescent girls who drank have higher odds of depressive symptoms, and adolescent mental health and alcohol programs should consider prioritizing this sub-group accordingly. Secondly, with regard to exposure measurement, the past-year, past-month and binge-drinking behaviors in this study were measured from self-reported history. Exposure is a function of intensity, frequency and duration, so our method of reporting ever vs. never behavior effectively collapsed levels of the measured constructs. More detailed measure, similar to how pack-year measures smoking, can allow for assessment of dose-response relationship and enhance the understanding of the association between drinking and depression. Thirdly, with regard to outcome measurement, the depressed mood question is subjected to inaccuracies. The association between drinking behaviors and clinical depressive disorder may be different from the association between drinking behaviors and depressed mood. Fourthly, although the participants were informed that the questionnaires were anonymous, the survey was conducted in the classroom setting with teachers in the vicinity. Social desirability could have influenced under-reporting of drinking behaviors, potentially biasing the measures of association toward null. The effect of social desirability in self-report of undesirable characteristics varies by age[47], thus potential biases in measurement of exposure and outcome also might have varied by sub-groups. Lastly, this study also has a limitation with regard to generalizability. Thailand has a relatively low secondary education attendance: in 2016, students in Years 7 thru 9 constituted 88.6% of the population age 12–14 years, and students in Years 10 thru 12 constitute 70.9% of the population age 15–17 years[48]. This study only included the population who were attending school, thus the results cannot be generalized to the Thai population not in secondary education.

## Conclusion

We found associations between alcohol consumption behaviors (past-year drinking, past 30 days drinking, and binge-drinking in past 30 days) and depressed mood among adolescent in all age groups and in both sexes. These associations were strongest among early-adolescent girls (those in Years 7 and 9) and weakest among middle-adolescent boys (those in Year 11), suggesting possible effect modification by age and sex. Early adolescent girls who drink should be considered at high likelihood of having depression, and adolescent girls who have depression should be considered at high likelihood of engaging in underage drinking. Future studies should consider assessing effect modification by school type.

## References

[1] Parekh R. What is Depression? In: American Psychiatric Association [Internet]. 1 2017 Available: https://www.psychiatry.org/patients-families/depression/what-is-depression

[2] World Health Organization. Adolescent health epidemiology. 2018 [cited 25 Dec 2018]. Available: https://www.who.int/maternal_child_adolescent/epidemiology/adolescence/en/

[3] Thapar A, Collishaw S, Pine DS, Thapar AK. Depression in adolescence. Lancet. 2012;379: 1056–1067. 10.1016/S0140-6736(11)60871-4 22305766 PMC3488279

[4] Maughan B, Collishaw S, Stringaris A. Depression in Childhood and Adolescence. J Can Acad Child Adolesc Psychiatry. 2013;22: 35–40. 23390431 PMC3565713

[5] Center on Alcohol Marketing and Youth. Prevalence of Underage Drinking. 2017. Available: http://www.camy.org/_docs/resources/fact-sheets/Prevalence%20of%20Underage%20Drinking_printer%20friendly.pdf

[6] Paileeklee S, Dithisawatwet S, Seeoppalat N, Thaikla K, Jaroenratana S, Chindet A, et al A Surveillance of alcohol, tobacco, substance use and health-risk behaviors among high school students in Thailand. Thai Health Promotion Foundation (ThaiHealth); 2016 9 p. 81.

[7] Jewpattanakul Y, Samai T. Thai Family Alcohol Consumption. J R Thai Army Nurses. 2014;15: 305–311.

[8] Thai Rath Newspaper. Tag: Depression. 2019 [cited 24 Aug 2019]. Available: https://www.thairath.co.th/tags/%E0%B9%82%E0%B8%A3%E0%B8%84%E0%B8%8B%E0%B8%B6%E0%B8%A1%E0%B9%80%E0%B8%A8%E0%B8%A3%E0%B9%89%E0%B8%B2

[9] Sobhonnarin S. Depression: A Common Disease or Just Something You Imagined. 3 Jan 2019 [cited 24 Aug 2019]. Available: https://www.phyathai.com/article_detail/2876/th/%E0%B9%82%E0%B8%A3%E0%B8%84%E0%B8%8B%E0%B8%B6%E0%B8%A1%E0%B9%80%E0%B8%A8%E0%B8%A3%E0%B9%89%E0%B8%B2_%E0%B9%82%E0%B8%A3%E0%B8%84%E0%B8%AE%E0%B8%B4%E0%B8%95%E0%B8%AB%E0%B8%A3%E0%B8%B7%E0%B8%AD%E0%B9%81%E0%B8%84%E0%B9%88%E0%B8%84%E0%B8%B4%E0%B8%94%E0%B9%84%E0%B8%9B%E0%B9%80%E0%B8%AD%E0%B8%87

[10] Thammasart University. Understanding Depression through the Eyes of a Psychologist. 29 Mar 2019 [cited 24 Aug 2019]. Available: https://tu.ac.th/thammasat-liberal-arts-psychology-depression

[11] Kongsuk T, Supanya S, Kenbubpha K, Phimtra S, Sukhawaha S, Leejongpermpoon J. Services for depression and suicide in Thailand. WHO South-East Asia J Public Health. 2017;6: 34–38. 10.4103/2224-3151.206162 28597857

[12] Wongpakaran T, Wongpakaran N, Pinyopornpanish M, Srisutasanavong U, Lueboonthavatchai P, Nivataphand R, et al Baseline characteristics of depressive disorders in Thai outpatients: findings from the Thai Study of Affective Disorders. Neuropsychiatr Dis Treat. 2014;10: 217–223. 10.2147/NDT.S56680 24520194 PMC3917918

[13] Department of Disease Control. Alcohol Beverage Control Act of B.E. 2551. Royal Gazette; 2008. Available: http://old.ddc.moph.go.th/law/showimg5.php?id=77

[14] Wichaidit W, McNeil E, Saingam D, Assanangkornchai S. Alcohol Consumption in Thai Society Report 2017. Hat Yai, Thailand: Center for Alcohol Studies; 2019 p. 150.

[15] Crews FT, Vetreno RP, Broadwater MA, Robinson DL. Adolescent Alcohol Exposure Persistently Impacts Adult Neurobiology and Behavior. Morrow LA, editor. Pharmacol Rev. 2016;68: 1074–1109. 10.1124/pr.115.012138 27677720 PMC5050442

[16] Edwards AC, Heron J, Dick DM, Hickman M, Lewis G, Macleod J, et al Adolescent Alcohol Use Is Positively Associated With Later Depression in a Population-Based U.K. Cohort. J Stud Alcohol Drugs. 2014;75: 758–765. 10.15288/jsad.2014.75.758 25208193 PMC4161696

[17] Marshall EJ. Adolescent Alcohol Use: Risks and Consequences. Alcohol Alcohol. 2014;49: 160–164. 10.1093/alcalc/agt180 24402246

[18] Patrick ME, Schulenberg JE. Prevalence and Predictors of Adolescent Alcohol Use and Binge Drinking in the United States. Alcohol Res Curr Rev. 2014;35: 193–200. 10.35946/arcr.v35.2.10 PMC3908711 24881328

[19] Boden JM, Fergusson DM. Alcohol and depression. Addict Abingdon Engl. 2011;106: 906–914. 10.1111/j.1360-0443.2010.03351.x 21382111

[20] Pedrelli P, Shapero B, Archibald A, Dale C. Alcohol use and depression during adolescence and young adulthood: a summary and interpretation of mixed findings. Curr Addict Rep. 2016;3: 91–97. 10.1007/s40429-016-0084-0 27162708 PMC4859438

[21] Kinyanda E, Kizza R, Abbo C, Ndyanabangi S, Levin J. Prevalence and risk factors of depression in childhood and adolescence as seen in four districts of North-Eastern Uganda. BMC Int Health Hum Rights. 2013;13: 19–19. 10.1186/1472-698X-13-19 23561039 PMC3626891

[22] MacPhee AR, Andrews JJW. Risk factors for depression in early adolescence. Adolescence. 2006;41: 435–466. 17225661

[23] Robkob W, Skulphan S, Sethabouppha H. Depression, Alcohol Drinking Behaviors, and Suicidal Risks of Adolescents. Nurs J (Manila). 2018;45: 144–158.

[24] Kroenke K, Spitzer RL, Williams JBW. The Patient Health Questionnaire-2: validity of a two-item depression screener. Med Care. 2003;41: 1284–1292. 10.1097/01.MLR.0000093487.78664.3C 14583691

[25] Richardson LP, Rockhill C, Russo JE, Grossman DC, Richards J, McCarty C, et al Evaluation of the PHQ-2 as a brief screen for detecting major depression among adolescents. Pediatrics. 2010;125: e1097–e1103. 10.1542/peds.2009-2712 20368315 PMC3100798

[26] Assanangkornchai S, Muekthong A, Inthanon T, editors. A Surveillance of Drinking Behaviors and Other Health Risk Behaviors among High School Students in Thailand. 1st ed. Hat Yai, Thailand: Research and Development Office, Prince of Songkla University; 2008 Available: http://kb.hsri.or.th/dspace/bitstream/handle/11228/2784/hs1619.pdf?sequence=3&isAllowed=y

[27] Boelema SR, Harakeh Z, van Zandvoort MJE, Reijneveld SA, Verhulst FC, Ormel J, et al Adolescent Heavy Drinking Does Not Affect Maturation of Basic Executive Functioning: Longitudinal Findings from the TRAILS Study. PLOS ONE. 2015;10: e0139186 10.1371/journal.pone.0139186 26489080 PMC4619383

[28] Koning IM, van den Eijnden RJJM, Verdurmen JEE, Engels RCME, Vollebergh WAM. A cluster randomized trial on the effects of a parent and student intervention on alcohol use in adolescents four years after baseline; no evidence of catching-up behavior. Addict Behav. 2013;38: 2032–2039. 10.1016/j.addbeh.2012.12.013 23391851

[29] Lumley T. Complex surveys: a guide to analysis using R. Hoboken, New Jersey: John Wiley & Sons, Inc; 2010.

[30] Chongsuvivatwong V. Analysis of epidemiological data using R and Epicalc. Hat Yai, Thailand: Epidemiology Unit, Prince of Songkla University; 2007.

[31] Assana S, Laohasiriwong W, Rangseekajee P. Quality of Life, Mental Health and Educational Stress of High School Students in the Northeast of Thailand. J Clin Diagn Res JCDR. 2017;11: VC01–VC06. 10.7860/JCDR/2017/29209.10429 28969248 PMC5620889

[32] Kaewpornsawan T, Tuntasood B. The Prevalence of Depression in 2nd Year High School Students in Bangkok. J Psychiatr Assoc Thail. 2012;57: 395–402.

[33] Jatchavala C, Chan SWY. Thai Adolescent Depression: Recurrence Prevention in Practice. J Health Sci Med Res. 2018;36: 147–155.

[34] Fillmore MT, Jude R. Defining “binge” drinking as five drinks per occasion or drinking to a .08% BAC: which is more sensitive to risk? Am J Addict. 2011;20: 468–475. 10.1111/j.1521-0391.2011.00156.x 21838847 PMC3156624

[35] Hasin DS, Grant BF. The National Epidemiologic Survey on Alcohol and Related Conditions (NESARC) Waves 1 and 2: Review and summary of findings. Soc Psychiatry Psychiatr Epidemiol. 2015;50: 1609–1640. 10.1007/s00127-015-1088-0 26210739 PMC4618096

[36] Johannessen EL, Andersson HW, Bjørngaard JH, Pape K. Anxiety and depression symptoms and alcohol use among adolescents - a cross sectional study of Norwegian secondary school students. BMC Public Health. 2017;17: 494–494. 10.1186/s12889-017-4389-2 28535753 PMC5442592

[37] Danzo S, Connell AM, Stormshak EA. Associations between alcohol-use and depression symptoms in adolescence: Examining gender differences and pathways over time. J Adolesc. 2017;56: 64–74. 10.1016/j.adolescence.2017.01.007 28167374 PMC5578443

[38] Cook MN, Peterson J, Sheldon C. Adolescent depression: an update and guide to clinical decision making. Psychiatry Edgmont Pa Townsh. 2009;6: 17–31. PMC2766285 19855857

[39] Uher R, McGuffin P. The moderation by the serotonin transporter gene of environmental adversity in the etiology of depression: 2009 update. Mol Psychiatry. 2010;15: 18–22. 10.1038/mp.2009.123 20029411

[40] Kongpraphan N, Trisit T, Chuaiple P. Quality of Life of Transgender Adolescents in Suphanburi. ARU Res J. 2019;6: 25–32.

[41] Nikratok P, Nilvarangkul K. Abused in Adolescent Katoey (Male Transgender). J Nurs Assoc Thail Northeast Div. 2013;31: 61–69.

[42] Kittiteerasack P, Sangngam J, Matthews AK. The Fundamentals of Child and Adolescent Nursing in Improving Mental and Psychosocial Health among Gender Variant Children in Thailand. J Nurs Sci. 2018;36: 4–16.

[43] Brière FN, Rohde P, Seeley JR, Klein D, Lewinsohn PM. Comorbidity Between Major Depression and Alcohol Use Disorder From Adolescence to Adulthood. Compr Psychiatry. 2014;55: 526–533. 10.1016/j.comppsych.2013.10.007 24246605 PMC4131538

[44] Clark DB, De Bellis MD, Lynch KG, Cornelius JR, Martin CS. Physical and sexual abuse, depression and alcohol use disorders in adolescents: onsets and outcomes. Drug Alcohol Depend. 2003;69: 51–60. 10.1016/s0376-8716(02)00254-5 12536066

[45] Sam-angsri N, Assanangkornchai S, Pattanasattayawong U, Muekthong A. Health-risk behaviors among high-school students in southern Thailand. J Med Assoc Thail Chotmaihet Thangphaet. 2010;93: 1075–1083. 20873081

[46] Wongtongkam N, Ward PR, Day A, Winefield AH. Student Perspectives on the Reasons for Physical Violence in a Thai Vocational College: An Exploratory Study. Deviant Behav. 2015;36: 187–199. 10.1080/01639625.2014.923277

[47] Soubelet A, Salthouse TA. Influence of social desirability on age differences in self-reports of mood and personality. J Pers. 2011;79: 741–762. 10.1111/j.1467-6494.2011.00700.x 21682727 PMC3139822

[48] Office of the National Education Commission. Education in Thailand 2018 Bangkok, Thailand: Office of the National Education Commission; 2018 12 p. 293 Available: https://pmnk.kkzone1.go.th/data/news3/24-02-2019-17-34-35_1344028011.pdf

